# Perception of integrating complementary and alternative medicine practice in patient’s treatment among the healthcare practitioners: a systematic review

**DOI:** 10.11604/pamj.2022.43.19.31133

**Published:** 2022-09-13

**Authors:** Azimatun Noor Aizuddin, Mohd 'Ammar Ihsan Ahmad Zamzuri, Juliana Mansor, Siti Rohani Nurumal, Sharifah Zawani Syed Ahmad Yunus, Mohamad Aznuddin Abd Razak, Mohd Nazrin Jamhari, Tong Seng Fah, Hazlina Mohd Miskam, Rozita Hod, Hanizah Mohd Yusoff

**Affiliations:** 1 Department of Community Health, Faculty of Medicine, National University of Malaysia, Kuala Lumpur, Malaysia,; 2 Department of Family Medicine, Faculty of Medicine, National University of Malaysia, Kuala Lumpur, Malaysia

**Keywords:** Complementary and alternative medicine, perception, healthcare practitioner

## Abstract

There is a growing trend in complementary and alternative medicine (CAM) usage among the population with medical conditions. However, there is hesitancy for medical practitioners to integrate its application with the current treatment modality, despite governance by the authority. Hence, our objective is to systematically evaluate the healthcare perception towards integrating CAM in their practices. We systematically searched three large and renowned databases i.e., Scopus, Web of Science and PubMed, regarding “Perception on Integrating CAM Usage in Patient's Treatment among Healthcare Practitioners” from 2016 until 2020. At least two independent reviewers comprehensively screened and extracted the data from the accepted articles. A total of 15 studies were included in the final qualitative synthesis following a strict and rigorous assessment checked using MMAT 2018 checklist. The studies included providing the richness of information due to the qualitative nature of the study design. There were three main domains extracted i.e. knowledge, attitude, and perspective of the healthcare practitioner towards CAM integration. Limited knowledge of CAM among healthcare providers may be the possible main reason for non-supportive attitude and negative perspective on CAM. However, those who showed an inclination towards CAM were found to be more open and ready to learn about CAM if it provides benefits to the patients. There is a heterogeneity of perception towards CAM integration from healthcare providers' point of view. A proactive and systematic CAM literacy awareness program may help to improve their understanding and possibly gain more trust in its application.

## Introduction

The world population is consistently facing health disease burden either from communicable or non-communicable disease. The practice of conventional medicine is the mainstream health system in most countries in treating diseases. However, in this current era, the treatment modalities are accessible within the spectrum of conventional medicine to complementary and alternative medicine (CAM). CAM is a general term referring to a broad field of medical “therapies” that is different from the conventional medical treatment practice in hospitals. According to the National Centre for Complementary and Integrative Health (NCCIH), there are five main groups of CAM, namely alternative medical system, mind-body interventions, biologically therapies, manipulative and body-based methods as well as energy therapies [1]. The NCCIH was founded in the year of 1998 in the United States by the National Institute of Health (NIH), which is responsible for the training, scientific research and disseminate information on CAM to patients as well the healthcare providers. CAM research aims to look at the effectiveness, safety and quality of CAM modalities that are available.

As reported in a systematic review, there was substantial CAM use (9.8% - 76%) among the general population in 15 countries surveyed [2]. Meanwhile, in Malaysia, it was estimated that the prevalence of CAM usage among Type 2 Diabetes Mellitus (T2DM) patients in primary care settings was 62.5% [3]. Another study that was conducted among cancer patients showed that the prevalence of CAM use was 70.2% with the most common types of CAM being used were biological-based therapies (90.2% and mind-body interventions 42%) [4]. On the other hand, another study found that the prevalence of CAM usage varied across diseases where 62.8% in cancer patients, 53.3% in hypercholesterolemia, 49.4% in hypertensive and 48.6% in diabetics [5].

At the same time, evidence had shown that conventional medicine has been steadily reducing morbidity and mortality as well increase the quality of life for the past decades in managing most of the disease. However, CAM practised were perceived to be more effective compared to conventional medicine based on the population survey conducted among elderly patients in Malaysia. Half of the respondents (55.1%) agreed, from a total of 256 respondents in the study [6]. The findings were the same in other studies conducted in India among patients at tertiary care hospital, where 50% out of the 403 patients believed CAM is more effective than conventional medicine [7]. On the other hand, other reasons given in the same study that adhered the patients to use CAM were less expensive, easily available, safer and felt better. At the same time, the majority of the patients also did not inform the use of CAM to the treating doctors. Previous studies showed only 60%, 42% and 19% revealed CAM usage to their doctors [7-9]. Among the reason given where CAM is not harmful and is not relevant to be informed [9]. Thus, these issues were considered as a threat if the patients' reliance on CAM is greater than conventional medicine. Furthermore, it can also cause unintended consequences if certain CAM modalities are combined with conventional medicine without the information of the treating doctors. In other ways, it is difficult to get the optimum health outcome as the overall treatment was not known by the doctors that provide care to their patient.

At the time being, there is a scarce study on CAM among healthcare practitioners. Therefore, this review aims to determine the perception of integrating CAM in patient's treatment among healthcare practitioners.

## Methods

**Search strategy:** this systematic review was conducted using three large and renowned databases, i.e. PubMed, Web of Science, and Scopus, regarding the “Perception on Integrating CAM Usage in Patient's Treatment among Healthcare Practitioners” from the year 2016 until 2020. This search was conducted in accordance with the Preferred Reporting Items for a Systematic Review and Meta-Analysis (PRISMA) checklist [10]. A table reporting all the article obtained was added as Annex 1 and PRISMA Checklist as Annex 2. The keywords used were as below and the search strategies as Annex 3: “Complementary medicine” OR “traditional medicine” OR “alternative medicine” AND “Belief” OR “perception” OR “perspective” OR “attitude” AND “Healthcare practitioner” OR “healthcare worker” OR “healthcare professional” OR healthcare personnel” OR “doctor” OR “physician” OR “medical assistant” OR “nurse” OR “pharmacist”.

**Figure 1:**
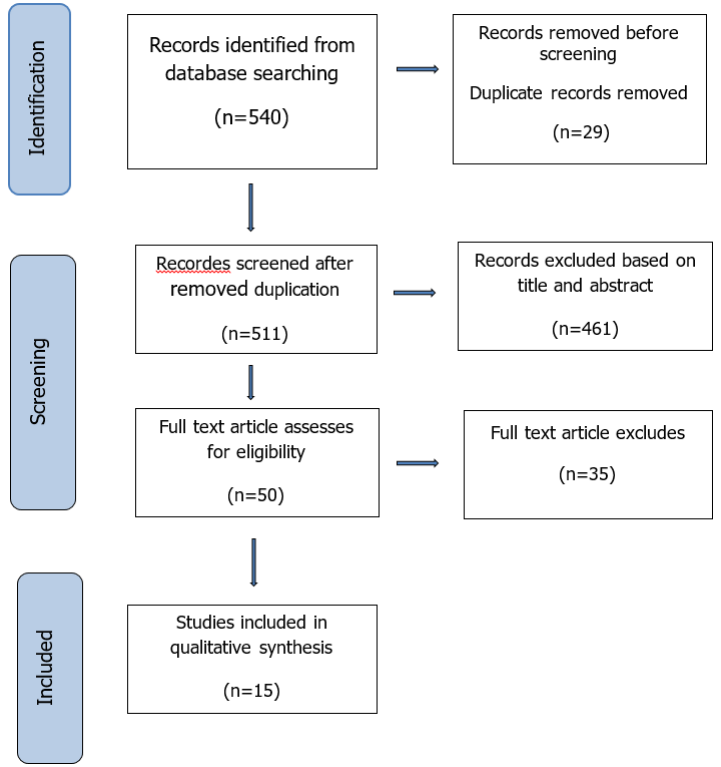
PRISMA flow diagram showing the selection for studies of perception on integrating CAM usage in patient's treatment among the healthcare practitioners

**Inclusion and exclusion criteria:** the target population of this search was any healthcare practitioner. The inclusion criteria from the database searches were (a) Original article (Qualitative), (b) All medical disease or advice, and (c) Availability of full-text article. The exclusion criteria in this search were based on (a) Quantitative type in design, (b) Systematic and narrative review paper article, and (c) non-English article. The articles were then identified through the titles and abstracts screening process according to the eligibility criteria. Full-text articles obtained were subsequently included in the qualitative synthesis. The flow of the article search is described in Figure 1.

**Data extraction tool:** all included articles were extracted by two independent authors and in case of inconsistency, a third author was consulted. The data was customized into (a) Number; (b) Year; (c) Author and Country; (d) Titles (e) Study Design; (f) Type of methods and analysis; (g) Result-themes generated; and (h) Conclusion.

**Operational definition:** the definition stated by NCCIH on CAM were simply as a group of diverse medical and healthcare interventions, practices, products, or disciplines that are not generally considered as part of conventional medicine. The healthcare providers include all practising professionals in the medical field who may have direct or indirect contact with the patient either by giving treatment or medical advice. They may include doctors, physicians, surgeons, nurses, medical assistance, occupational therapist, physiotherapist, pharmacist etc.

## Current status of knowledge

### Results

A total of 511 articles were initially obtained for the title and abstract screening, while only 50 articles were left for full-text screening. The common reason for omitting the other 35 articles upon full-text review was due to the study design, different objectives, not the target population, and not related to CAM. Two reviewers assessed the quality independently using the mixed method assessment tool Mixed Method Assessment Tool (MMAT) version 2018. Articles were only selected if both reviewers agreed with the quality. Any disagreement between the assigned reviewers will employ a third independent reviewer. All the included studies answered “yes” for all the questions of the respective domains of MMAT checklists which are risk of bias assessment that is present as Annex 4.

The distribution of the articles varies with four articles from Asia (Indonesia 2, Iran 1 and Saudi Arabia 1); three articles each from the USA and Australia; two articles each from the United Kingdom and Germany; and one paper from Ghana.

**Figure 2:**
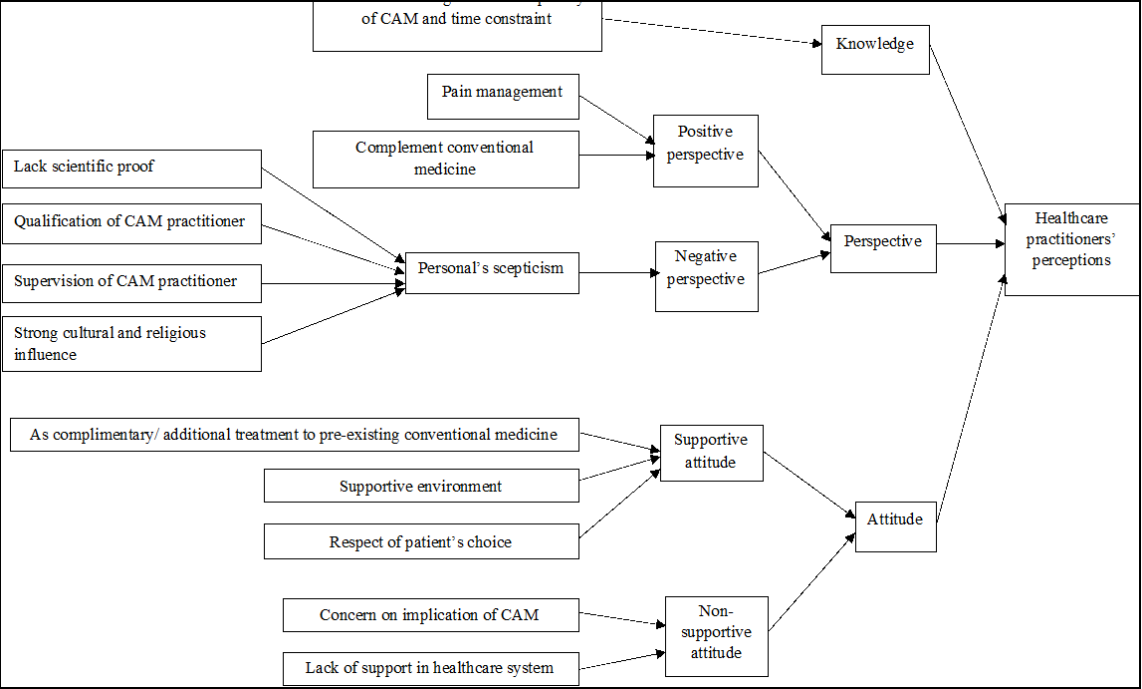
factors affecting the practitioner's perception in integrating CAM

The findings can be broadly categorized into three main domains which are knowledge, perspective, and attitude towards CAM by the healthcare provider. Due to the richness of the data from qualitative type of research, some of the themes overlap with one another. Therefore, two of our domains which are perspective and attitude were further broken down into more sub-themes and categories. Factors affecting the practitioner's perception in integrating CAM are described Figure 2.

**Domain (*knowledge):*** overall, studies that looked into this domain reported the lack of knowledge in CAM among medical practitioners [11-13]. According to Kretchy *et al*. [14], almost all the professions that were sampled showed limited knowledge and mostly knew and perceived CAM to be associated with herbal medicine only. Among the possible reasons for the lack of knowledge on CAM found by Wardle, Sibbritt and Adams [15] were the complexity of CAM treatment and the time constraint in learning about CAM that was faced by the medical practitioners. Sharp *et al*. [16] suggested the policy maker to lay out a clear theme and objective if they require the medical practitioners to improve their knowledge and education about CAM.

**Domain: *perspective***

***Positive perspective:*** there are two sub-themes derived in this domain, which are positive perspective and negative perceptive. The integration of CAM with conventional treatment have received two types of fates, either being positively accepted or the idea clearly being denied by the medical practitioner. To put in highlight, the reason for positive acceptance by the healthcare worker was the probable good additional impact that CAM could contribute to the treatment. This is especially true in pain management as most of the practitioners who have a positive perspective on CAM echoing its usage as an additional modality. A study by Sharp *et al*. [16] on patients with chronic Musculoskeletal (MSK) pain employed CAM due to the limited conventional treatment that they can offer. The same scenario was also faced by Penney *et al*. [17] that utilized opioid-based analgesia in the management of chronic pain where they ended up incorporating CAM in the treatment. On the other hand, Shannon *et al*. [18] claimed that the integration of CAM in the treatment of rehabilitative patient will not only help to improve pain management but also fasten the time for discharge, improved functional capacity and increase the Activity of Daily Living (ADL). An interesting finding by Liem [19] stated that the additional benefit impact of CAM treatment was due to some good personal experience in treating their own pain. In addition to improving pain management, another reason for the positive perspective towards CAM integration among medical practitioners was the modality that complements with others. Mollart, Adams and Foureur [20] described that CAM treatment (acupressure) on post-date pregnancy complements the wellness model of pregnancy and childbirth. The midwife claimed that the method helps in normalizing pregnancy and birth as it releases stress and instill good inner motivation.

***Negative perspective:*** the opposite sub-theme that emerged was the negative perspective on CAM integration. One of main the reason for the negative perspective towards CAM was the skepticism towards it despite patients claiming of having a good result after using it. According to Christina *et al*. [13], nurses who work in the oncology unit in Indonesia were skeptical with CAM treatment on cancer patients. They agreed not to integrate CAM with conventional management. Nevertheless, Corina, Christine and Klein [21] found that even the oncologists were skeptical in CAM as it lacks apparent scientific proof to ascertain its use. The study by Sharp *et al*. [16] also found that some of the general practitioners were skeptical on CAM in treating MSK pain with mental health comorbidity. Jarvis *et al*. [22] explained in their study that the skepticism on CAM came from the qualification of CAM practitioners themselves. He also queried the existence of a regulatory body that supervised CAM's practices. In another finding, the medical practitioners were skeptical with CAM as they employed misleading advertisement to picture the good outcome of using it despite being an irrational and ineffective treatment [19]. Becker *et al*. [12] on the other hand, stated in its finding that the non-pharmacologic treatments (including chiropractic treatment) were sub-standard and not effective in treating chronic pain patients.

Other than being skeptical, another reason for the negative perspective on CAM was the modality that has some link with cultural and religious belief. For example, Corina, Christine and Klein [21] in their finding revealed that those medical practitioners who were not fond of CAM described it as belonging to another world, which almost certainly not able to be scientifically proven. In addition, some Muslim's oncologist and medical scholars in Saudi Arabia refuse to open Pandora's box by utilizing CAM on cancer patient as they believe treatments should be evidence-based and not simply according to religious affiliation [23].

**Domain: *attitude***

***Supportive attitude:*** the sub-theme of supporting attitude towards CAM can be further subdivided into three categories. One category that supports the integration was based on its true definition of being a complementary or supporting treatment. Anheyer *et al*. [11] found that both the doctors and nurses were in favour to integrate CAM with the mainstream treatment in the paediatric clinical setting. Some of them even go beyond by recommending it not to be left out when deciding the treatment at the first point of contact. Alqahtani *et al*. [23] also added that some healthcare providers believe that CAM can be incorporated as a holistic aspect in cancer care without jeopardizing the conventional treatment. Again Penney *et al*. [17] showed a positive attitude towards CAM as it helps patients to achieve short term pain relief, hence, reducing the reliance on opioids. Kretchy *et al*. [14] supportive attitude towards CAM because they viewed it as an alternative form of care that augment the challenges associated with allopathic care.

The positive attitude towards CAM was also shown in the sub-theme of displaying interest in the intervention. Tagharrobi, Mohammadkhan Kermanshahi and Mohammadi [24] showed that given a supportive environment and greater opportunity of practising CAM, nurses at the critical care unit were more likely to have a positive attitude towards using CAM. This phenomenon was also seen in the study by Anheyer *et al*. [11] where all of the respondents showed interest to learn about CAM as the setting favour the integration of modalities. Findings from Corina, Christine and Klein [21]; Christina *et al*. [13] showed that despite having scepticism towards CAM, the healthcare providers were still willing and show openness to learn about it.

Another reason for the supportive attitude on CAM by medical practitioners was due to the respect of patient's choice. Liem [19] highlighted in his finding that patient's choice needs to be respected if they voice request for alternative treatment. This is exactly in line with a study by River *et al*. [25] that demonstrated the priority of person-centred care and to allow for patient's care preferences, especially in cancer care management. Moreover, Tagharrobi, Mohammadkhan Kermanshahi and Mohammadi [24] extended the finding by introducing the concept of consumer demand in association with respect for patient's choice. They found that through client's questioning, client's requests, and even complaints about lack of CAM have led to the supportive attitude towards CAM.

***Non-supportive attitude:*** on the other hand, the sub-theme of non-supportive attitude towards CAM can be categorized into two; due to concern of implication and from the lack of support in healthcare services. Anheyer *et al*. [11] raised the concern of possible allergies and side effect of CAM in clinical care. Without proper immediate management available at the setting, healthcare practitioners were less likely to show support on CAM. Kretchy *et al*. [14] and Liem [19] highlighted the possible illness complications as a result of a delay in formal help-seeking behaviour and strong non-adherence to the medical advice and treatment that made medical professionals less supportive of CAM. Wardle, Sibbritt and Adams [15] were concerned about the CAM negative implication towards health because of its pseudoscientific nature and has no evidence base knowledge.

Another category, which is lack of support in healthcare services was found to be a non-supportive attitude towards CAM. This reason was mentioned in studies by Kretchy *et al*. [14] and Becker *et al*. [12]. Apart from that, financial resources have always been a challenge to develop CAM. According to Jarvis *et al*. [22], the lack of funding was associated with the lack of actual demand by the patients.

## Discussion

The interest in CAM has dramatically increased over the past years. In the UK, there was a high prevalence of herbal medicinal products that were bought over the counter, mostly self-prescribed. Based on our findings of the positive perspective subdomain, several articles reported the use of complementary and alternative medicine, such as chiropractic and acupuncture due to the reasoning of having an additional impact on the treatment and whole-person healing [18,20]. This additional benefit of CAM treatment was also seen in other literature, such as in the systematic review by Frass *et al*. [26] that highlighted the most common reasons for complementary and alternative medicine utilization, which were back pain problems, depression, insomnia, severe headache or migraine and stomach or intestinal illness. Nevertheless, another profound reason for using CAM is the desire to contribute to the treatment process and to improve general health [27].

With the above-mentioned positive perspective of benefit, it is not surprising for some medical practitioners to have a supportive attitude towards CAM. A study in India found that more than half of doctors working in tertiary hospital utilized CAM therapies and the most commonly utilized therapy was Homeopathy [7]. The doctors also believe in the beneficial role of CAM and recommended CAM as a therapy to their patients. While in Switzerland, healthcare professionals agreed that Complementary Medicine (CM) could be useful for the treatment of chronic pain and they recommended acupuncture to their patients if they had migraine, tension headache and low back pain [28]. This study also found that 96.9% were strongly in favour or in favour of offering CM, especially hypnosis (89.8%), osteopathy (85.5%), and acupuncture (83.4%) at the hospital for treating chronic pain.

Another noted reason for medical practitioners to have a supportive attitude towards CAM was respect for patient's choice. Respecting patient's choice is one of the arts in treating disease. It shows effective doctor-patient communication, especially in giving support to patients to find hopes in curing their diseases. According to Kelak, Cheah and Safii [29], doctor's interpersonal and communication characteristics of being involved, treating patients respectfully, listening attentively, respecting privacy, and leaving time for the patient are critical components for patient's disclosure in choosing CAM and contribute to patient's positive attitude to continue their treatment. Doctors who respect their patient's choice in choosing CAM are usually among those who are knowledgeable and aware about CAM. They will inform their patient about the conventional treatment and other supplementary treatment that can help in treating their patients [25]. Enhancing patients' expectations through positive information about the treatment or illness, while providing support or reassurance, may influence health outcomes. A recent study among complementary therapy users who are cancer survivors suggested that effective communication may lead to the decision to use complementary therapy as a supplement rather than an alternative to conventional medicine [30].

Although our subdomain religion showed a negative perspective among medical practitioners towards CAM, many other studies found otherwise. A review by Alrowais and Alyousefi [31] noted religious modalities as the most type of CAM used among the Saudis. It is the most popular method of CAM among Middle East citizen. Apart from Quranic recitation, supplication, or consumption of Zamzam water or water where the Quran has been read upon, and black seeds are also practised. These were found to be the most commonly used by oncology patients in a Riyadh study, although, only 7.4% of the participants attributed their disease improvement to purely CAM use [32]. Nevertheless, other parts of the world also practise spiritual and religious method as CAM. Although, it varies based on religion, gender, age, and education, as well as the diseases. Although the true effectiveness of the modalities is still inconclusive, many patients opt to use them due to the perception of positive effects on health, sense of well-being, controlling the disease, cost-effective, easy access and improving the quality of life. Furthermore, it was shown that healthcare practitioners have a fair knowledge and positive attitude towards this modality [33]. Interestingly, a study in Trinidad and Tobago found that the knowledge on the spiritual or religious type of CAM was fair among the healthcare personnel. Only half of them openly discussed the use of CAM with their patients and only 15% were willing to refer their patients to CAM practitioners. But, a number of them seem to perceive the combination of CAM and conventional medicine more than conventional medicine alone for the patient's treatment [34].

We also found that medical practitioners have negative a perception towards CAM is because of their skepticism towards it. In a study conducted among the oncologist in Brazil, some of the participants had negative views on CAM due to the limitation of the resources in the healthcare system, thus, they need more evidence-based medicine for practice [35]. On the other hand, a study conducted among the same population of physician mentioned that they became more skeptical on CAM after witnessing the adverse effect when patients combined it with the conventional treatment [36]. Apart from that, another literature found that the lack of treatment effect of CAM made the physician less satisfied in using Chinese Medicine [37]. This will ultimately lead to growing skepticism towards CAM by the medical personnel.

## Conclusion

Our systematic review showed a mixture of perception among healthcare practitioners in the integration of CAM in patients' management that were presented into three main domains and respective subdomains. Many factors were highlighted, ranging from personal experience until the concern of implication if CAM superseded conventional treatments. Nevertheless, knowledge on CAM still remains low among healthcare providers. More awareness program, targeting medical professional, is needed, to successfully integrate CAM in patients care.

### What is known about this topic

- CAM among healthcare practitioners is a new body of knowledge although it is widely practice in some population;
- Some patients used CAM as a replacement modality to treat their chronic non-communicable diseases;
- The integration of CAM together with conventional medicine is limited due to lack of safety and efficacy data.

### What this study adds

- The study has highlighted a low level of knowledge about CAM and its limited application among the healthcare practitioners;
- The heterogeneity of perception regarding integration of CAM modality with conventional treatment hinders its application by the healthcare practitioners;
- Healthcare practitioners' attitude towards CAM can skewed towards acceptance as a result of environment and patient factors.

## Annexes

**Annex 1**: summary of the articles obtained (PDF, 364 KB)

**Annex 2**: PRISMA checklist (PDF, 342 KB)

**Annex 3**: search strategies (PDF, 319 KB)

**Annex 4**: Mixed Methods Appraisal Tool (MMAT) for risk of bias assessment (PDF, 357 KB)
